# The Effects of mmW and THz Radiation on Dry Eyes: A Finite-Difference Time-Domain (FDTD) Computational Simulation Using XFdtd

**DOI:** 10.3390/s23135853

**Published:** 2023-06-24

**Authors:** Negin Foroughimehr, Zoltan Vilagosh, Ali Yavari, Andrew Wood

**Affiliations:** 1 6G Research and Innovation Lab, Swinburne University of Technology, Melbourne, VIC 3122, Australia; zvilagosh@swin.edu.au (Z.V.); mail@aliyavari.com (A.Y.); awood@swin.edu.au (A.W.); 2 School of Health Sciences, Swinburne University of Technology, Melbourne, VIC 3122, Australia; 3 School of Science, Computing and Engineering Technologies, Swinburne University of Technology, Melbourne, VIC 3122, Australia

**Keywords:** electromagnetic bioeffects, cornea, finite-difference time-domain, FDTD, 5G radiation

## Abstract

The importance of investigating the health effects of RF radiation on the cornea cannot be overstated. This study aimed to address this need by utilizing a mathematical simulation to examine the absorption of millimeter wave (mmW) and terahertz (THz) waves by the cornea, considering both normal and pathological conditions. The simulation incorporated variations in tear film thickness and hydration levels, as these factors play a crucial role in corneal health. To assess the impact of RF radiation on the cornea, the study calculated temperature rises, which indicate heating effects for both dry and normal eyes. XFdtd, a widely used commercial software based on the Finite-Difference Time Domain (FDTD) method, was employed to evaluate the radiation absorption and resulting temperature changes. The outcomes of this study demonstrated a crucial finding, i.e., that changes in the water ratio and thickness of the tear film, which are associated with an increased risk of dry eye syndrome, directly impact the absorption of mmW and THz waves by the cornea. This insight provides valuable evidence supporting the interconnection between tear film properties and the vulnerability of the cornea to RF radiation.

## 1. Introduction

The millimetre wave (mmW) spectrum begins at 30 GHz and extends up to 300 GHz, corresponding to the wavelength range of 1–10 mm in the air on the electromagnetic (EM) spectrum [1]. The terahertz (THz) region is the portion of the EM spectrum ranging from 0.1 THz to 10 THz, corresponding to wavelengths of from 3 to 0.033 mm. At its lower-frequency end (0.1 THz), the THz region crosses over with mmWs, with an upper boundary of around 0.3 THz [2]. The presence of three low-atmospheric-absorption window frequencies, at 94 GHz, 140 GHz, and 220 GHz, provide an opportunity for wide-band, long-haul 5G wireless communication systems [1]. The 5th-generation technology (5G) is currently utilising the mmW band of 24–86 GHz [1]; future 6G technology is expected to revolutionize user experiences and enable the various innovative applications that have emerged with 5G. As we look toward the future of 6G devices, there is a growing emphasis on harnessing the potential of the EM spectrum’s THz region, specifically within the range of from 250 GHz to 4 THz [3].

Device-to-device (D2D) communication enables short-range direct communication without support from any existing infrastructure. The data rate of D2D communication can be significantly enhanced with the help of mmW technology. The 6th-generation (6G) cellular system is currently at the very early stages of its development and is expected to introduce an even higher data rate and lower latency to meet the demand of the Internet of Things (IoT). THz frequency is one of the candidates to enable ultra-high data rates in future 6G networks [4]. The power attenuation from 0.13 to 0.33 THz has low loss in the atmosphere; hence, this frequency bandwidth will be a reasonable choice for applications such as communications, sensing, and imaging [5]. With the increasing use of mmW and THz radiation in communication and medical applications, the potential impacts of mmW and THz technologies on biological systems become critical [6]. As 5G technology continues to advance and the possibility of 6G operating systems emerges, there is an increasing interest in utilizing higher frequencies of up to 4 THz on the EM spectrum [3]. RF fields exhibit decreased penetration into biological tissue as the radio frequency increases. Specifically, for frequencies surpassing 6 GHz, the penetration depth becomes relatively shallow [7], resulting in an EM energy deposition within superficial tissues, such as the cornea.

In recent years, several studies have utilized numerical simulations to investigate the thermal effects of EM radiation on the human eye. Various factors influencing the specific absorption rate (SAR) and temperature elevation in the eye due to EM exposure have been examined. These factors include eye size, as demonstrated in male and female Japanese eyes by Hirata et al. [8], palpebral fissure features investigated by Diao et al. [9], the impact of aqueous humor flow studied by Flyckt et al. [10], changes in axial ocular length explored by Li et al. [11] and the effect of grid resolution examined by Laakso [12]. The primary emphasis of the studies was to examine how EM radiation affects the tissues of a healthy eye. However, the scientific literature concerning the effects of RF energy on specific pathological eye conditions, like dry eyes, is limited. Additionally, there is a notable lack of research on computational modeling concerning the impact of high GHz and THz radiation on ocular tissues.

National and international standards and guidelines have been established to regulate and mitigate the potential risks associated with non-ionizing radiation (NIR). Organizations such as the International Commission on Non-Ionizing Radiation Protection (ICNIRP) and the World Health Organization (WHO) have provided recommendations for limiting exposure to electromagnetic (EM) fields. In Australia, the Australian Radiation Protection and Nuclear Safety Agency (ARPANSA) serves as the national authority responsible for radiation protection and nuclear safety, ensuring consistent policies and practices. The primary reference source for computational investigations on RF fields conducted in this study is the updated 2021 ARPANSA guideline [13]. This guideline, derived from the ICNIRP recommendations in 2020, serves as a key resource for assessing and addressing the potential effects of EM fields in relation to radiation protection.

Dry eye is a prevalent condition affecting individuals globally, resulting in ocular discomfort, a diminished vision quality, and interference with daily activities [14]. With the prevalence of dry eye disease ranging from 5 to 50 percent [15], understanding the impacts of mmW and THz radiation becomes even more crucial.

The present study is focused on the thermal effects of mmW and THz waves on the human cornea. The scope of this study includes the frequency range of (30 GHz–0.5 THz), which is a span of over one order of magnitude. The current study examines how changes in tear film thickness and hydration affect the absorption of mmW and THz radiation on the cornea.

The response of biological tissues to mmW exposure depends on their electric and magnetic properties at the exposure frequency. Rhe magnetic properties of a material are described by a complex permeability (μ), as shown in Equation (1).

(1) $$\mu =\mu^{\prime }-j\mu^{\prime \prime }$$

where μ represents the complex permeability, $\mu^{\prime }$ shows the real part of the permeability, which determines the material’s ability to respond to magnetic fields, and $\mu^{\prime \prime }$ indicates the imaginary part of the permeability, which accounts for the material’s magnetic losses.

Biological non-ferromagnetic tissues have a permeability that only slightly differs from the permeability of free space ($\mu_{0}$), and therefore, $\mu \approx \mu_{0}=4\pi \cdot 10^{-7}$ H/m. The electric properties of tissues can be characterised by the frequency-dependent complex permittivity (Equation (2), where $\varepsilon^{\prime }$ is the real part (dielectric constant) and $\varepsilon^{\prime \prime }$ is the imaginary part (loss factor).

(2) $$\varepsilon^{*}=\varepsilon^{\prime }-j\varepsilon^{\prime \prime }$$

The eye is susceptible to thermal damage due to the absence of blood flow in certain areas, such as the cornea and parts of the internal structure like the lens [16]. In the eye’s anterior chamber, heat dissipation occurs primarily through convection from the cornea’s surface and conduction through the sclera and lens [16]. A three-layer tear film protects the cornea’s surface, which serves as a crucial barrier against direct exposure to the surrounding air. In the context of EM exposure, the thermal ocular effects are anticipated to be more pronounced in individuals with dry eyes. This is attributed to the thinner tear film in such individuals, allowing for easier EM wave penetration [17]. SAR, measured in watts per kilogram (W/kg), quantifies the power absorbed per unit mass, which serves as a metric for evaluating tissue exposure and potential health risks, with specific values established for various frequencies and tissue types [18]. SAR is defined using Equation (3).

(3) $$\mathrm{SAR}=\frac{\sigma {\left|E\right|}^{2}}{\rho }$$

where σ is the electrical conductivity in S/m, and ρ is the material density (defined in kg/m^3^). This formula allows for SAR to be determined in watts per kilogram (Wkg^−1^).

Under conditions where heat loss due to conduction or blood flow is not significant, SAR and temperature rise are directly related, as follows [18].

(4) $$\mathrm{SAR}=C\frac{dT}{dt}$$

where *C* is the specific heat capacity of the tissue, *T* is the temperature (°C), and *t* is the exposure period. Equation (4) is useful for a short exposure period where heat loss is insignificant. The penetration depth (δ) of EM radiation into biological tissue defined by E-field strength ($\hat{E}$) is shown by Equation (5).

(5) $$E_{Tissue}\left(\delta \right)=\frac{1}{e}{\hat{E}}_{Tissue}\left(0\right)$$

where $E_{Tissue}\left(\delta \right)$ represents the E-field within the tissue at a particular depth/distance (δ). The penetration depth of biological tissue at 0.1 THz is in the order of from 1.0 to 1.5 mm and reduces with higher frequencies to ≈0.01 mm [19]. The human cornea contains 75–80% water. Two natural states of water in biological tissues are known as free “bulk” water (i.e., freely moving water molecules) and bound water [6,20]. Bound water has a lower absorption coefficient than bulk water at THz, which needs to be considered when calculating penetration depth [21]. The absorption coefficient of water is high compared to other components of biological tissue at mmW [22,23], which makes corneal water content the leading influence in cornea THz absorption. This study introduces a detailed and comprehensive three-dimensional (3D) model of the human eye, specifically developed to investigate the effects of MMW and THz radiation on the cornea in individuals with dry eye conditions. The developed FDTD model, extensively described in the subsequent sections, aims to address and bridge the existing gaps in the literature.

## 2. Material and Methods

In the following sections, the details of our numerical simulations, including the geometrical and material models, are introduced.

### 2.1. The Details of the Computational Model

The widely used finite-difference time domain (FDTD) method was utilised to solve the absorbed mmW and THz wave inside corneal tissues [24]. The main computational platform employed in this study was XFdtd (Bio-pro, Remcom, State College, PA, USA), a commercial software. XFdtd has gained widespread usage in numerous research studies focused on investigating the interaction between EM radiation and diverse biological tissues. Its extensive application in this field highlights its effectiveness and reliability in analyzing such phenomena [25,26,27,28,29,30]. For this particular study, the eye model underwent substantial enhancements compared to our previously developed model [31]. The subsequent section provides comprehensive descriptions of the anatomical features and dimensions of the model.

#### 2.1.1. Establishments of Anatomical Features of Simulation Model

The advantage of the anatomical phantom model used in the previous study [31] is that the dimensions of individual components can easily be altered, which allows for variations in the thickness of the tear film to be studied. The ocular surface is the most environmentally mucosal surface, and the tear film protects the ocular surface against irritants, dryness, environmental extremes of temperature, etc. The tear film comprises an inner mucous layer, a middle aqueous layer and a top oil layer [32]. The lacrimal gland is the main contributor to the tear film’s aqueous layer [14]. The tear film is assumed to comprise a single aqueous layer covering the cornea to reduce the computational model to a manageable complexity.

The tear film thickness is typically measured by interferometry. The thickness of the tear film is roughly about 6.0 µm ± 2.4 µm in normal subjects and is significantly thinner in dry eye patients with measured values as low as 2.0 µm ± 1.5 µm [32]. To address the inherent variability within each group, our model incorporates average tear film thickness values of 8.0 µm for normal subjects and 3.0 µm for dry eye patients. These values are chosen to approximate the mean values reported in the literature and to encompass the range of tear film thickness within each group, considering the provided standard deviations.

In XFdtd, the physical parameters, including the water content of the tissue, can be specified, and any materials that do not have a water ratio specified will be treated as if they have no water content. To simulate dry eye conditions, the water ratio of the tear film was set at 40%, and for a normal subject, the water ratio was set at 80%. This decision arises from the theoretical argument that the ratio of tear evaporation in total tear flow is increased compared with that of normal subjects [33].

The cornea is the human eye’s dominant optical element of the human eye providing about 75–80% of the refractive power [34]. In this model, the cornea is assumed to lie above the anterior chamber as a hemispherical shell with a constant diameter and thickness of 12 mm and 0.5, respectively. Neither blood vessels nor lymphatic vessels are found within the normal central cornea. Thus, this structure has no assigned blood perfusion rate in the current model.

In the human eye, the anterior chamber is filled with a clear nutritive fluid (i.e., the aqueous humour) that meets the metabolic needs of the avascular structures bordering the anterior chamber, including the cornea and the lens. In the presented model, the anterior chamber is modelled to be bounded anteriorly by the cornea and posteriorly by the iris and filled with aqueous humour.

**Figure 1:**
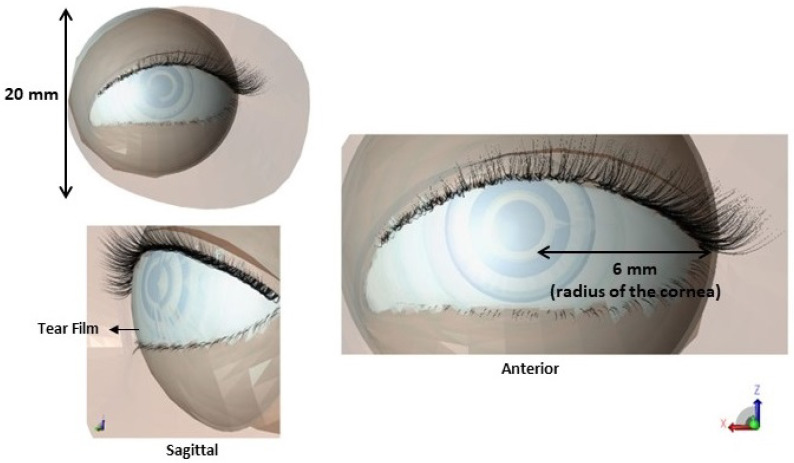
Constructed model of the eyeball in XFdtd.

**Figure 2:**
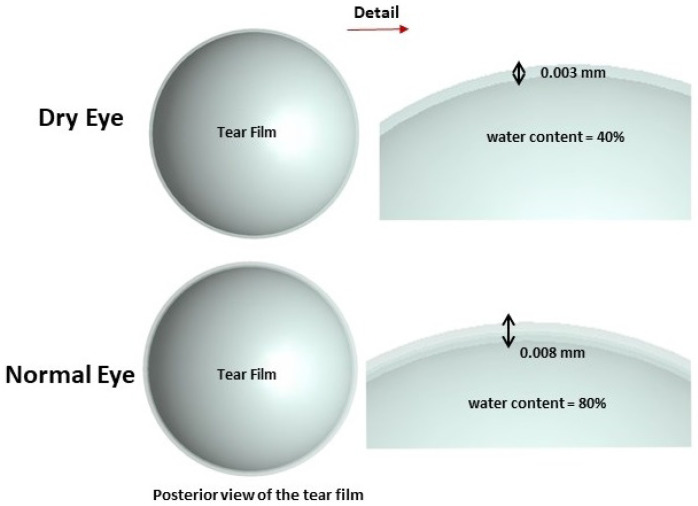
Dimensions and physical parameters of tear film in dry and normal eyes.

The size of the pupil and the amount of light reaching the retina is controlled by the iris [35]. Given that the blood flow inside the iris is relatively small compared to the blood flow inside the choroid, the thermal model presented in this study has ignored the presence of blood flow in this space. The skin of the eyelid is exceptionally thin, measuring less than 1 mm in thickness [32]. The skin is modelled with a single layer (i.e., epidermis). Figure 1 displays our designed eyeball; additionally, Figure 2 provides a detailed diagram showcasing various tear film thicknesses. It should be noted that all the structures in the 3D model used in this study were created by ourselves; however, to improve the model’s realism, we incorporated the “physical geometry” of the eyelids and eyelashes from a pre-existing model developed by Robbin Pittelkow (https://www.cgtrader.com/3d-models/character/anatomy/human-eye-with-eyelids, accessed on 19 December 2022).

#### 2.1.2. Simulation Environment in Remcom XFdtd

To effectively absorb the outgoing wave, perfectly matching layers (PMLs) were utilised as absorbing boundary conditions in all simulations. Steady-state electric field (E-field) and magnetic field (H-field) data were recorded using a Sine waveform at frequencies of 30 GHz, 60 GHz, 90 GHz, and 0.5 THz. The simulation convergence threshold was defined as −40 dB.

It is important to note that XFdtd generates the mesh by assigning magnetic and electric material properties to each Yee cell face and edge, respectively. This is achieved by analyzing the intersections between the grid and the geometry in the project and applying the corresponding materials to the appropriate Yee cell edges and faces. A detailed description of the simulation parameters can be found in Table 1. The Grid Editor in XFdtd provides essential controls for configuring the calculation grid, which is crucial for achieving accurate results and managing memory and runtime requirements. An optimal grid setup balances geometric details’ resolution and RF propagation modeling accuracy without excessive refinement. While increasing the grid resolution improves accuracy, it also increases simulation runtime and memory usage. The Project Optimized Gridding (PrOGrid) feature in XFdtd simplifies the determination of ideal balance between accuracy and runtime by automatically defining the FDTD grid. PrOGrid follows recommended practices for gridding, such as utilizing appropriate cell sizes for the simulation’s frequency range, enhancing grid resolution in high-permittivity tissues, accurately representing small geometric features using multiple grid cells, applying free space padding based on the largest resonant wavelength and incorporating smaller grid cells near discrete sources and good conductors. This ensures that the grid meets the desired computational efficiency and accuracy trade-off. The solver’s performance was previously verified using different meshing size techniques [31]. The accuracy of the FDTD solver is subject to the discretisation of cells depending on the ratio of cell resolution and wavelength (λ). A rule of thumb value of λ/10 is considered for the maximum applicable cell size [12]. We defined smaller FDTD grid cell sizes to maintain sufficient accuracy and provide adequate geometry resolution (i.e., λ/20). The capacity of the computer hardware limited the rendering of the anatomical detail of the eyeball. Hence, larger cell sizes were considered for simulations at 500 GHz (i.e., λ/12).

On a regular desktop computer, the estimated duration for a single simulation with the given configuration (including only one sensor) was approximately 9 h. However, leveraging the cloud computing environment, specifically the National eResearch Collaboration Tools and Resources Platform offered by the Australian Research Data Commons, resulted in a remarkable reduction in computation time to just 30 min.

#### 2.1.3. Dielectric Properties

As mentioned above, we developed a human eye model composed of multiple tissues. To integrate these structures into the XFdtd scheme, we need to define their dielectric properties. Dielectric values and thermal parameters of tissues were based on the values presented in [36,37]. The dielectric property of the tear film is assumed to be identical to the aqueous humour. The thermal properties of the biological material present in the model are shown in Table 2.

Due to the unavailability of dielectric data for ocular tissues such as the lens and the iris at a frequency of 0.5 THz, these components were excluded from the study. The dielectric properties for the cornea and the eyelids (skin) in the THz region were obtained from references [37,38]. Since the dielectric properties of aqueous humor closely resemble pure water at frequencies above 13 GHz [39], pure water was used as a substitute for aqueous humor in the THz region.

### 2.2. Exposure Scenario and Sensors

The cornea’s curvature affects the direction and distribution of the RF radiation when it reaches the corneal surface. Since RF radiation is typically emitted as plane waves, the exposure will be orthogonal (perpendicular) to the cornea’s surface on a small region that aligns with the curvature. This means that the RF radiation will interact with the cornea differently across its curved surface compared to a flat surface. To account for this interaction, the simulation in this study considered a plane wave polarized in the sagittal plane, specifically targeting the right side of the eyeball. This approach considers the cornea’s curvature and ensures that the RF radiation interacts with the cornea in a manner consistent with its natural anatomical shape. By simulating the exposure in this way, the study aims to provide a more accurate representation of the interaction between RF radiation and the cornea, considering the influence of curvature on the exposure distribution. The recent ICNIRP safety recommendation [18] for local power density for the frequency range of from >6 to 300 GHz for an average of 6 minutes’ exposure time is 100 Wm^−2^ and 20 Wm^−2^ for workers and the general public, respectively [18]. In this study, we considered a plane wave as a radiation source for all the simulations. Two incident power densities were studied using a thermal sensor: power density of 20 Wm^−2^ and 100 Wm^−2^, corresponding to maximum permissible exposure by ICNIRP [18]. For simulations involving the thermal sensor at frequencies of 0.5 THz and 1 THz, the estimated total system random-access memory (RAM) requirements for central processing unit (CPU)-based simulation(s) were 8 TB and 14 TB, respectively. The estimated time for a single simulation was approximately 10 days. Due to these resource constraints, temperature rise calculations were only performed up to 90 GHz.

## 3. Results

In the subsequent sections, we present the results of exposing the eyeball to a plane wave ranging from 30 GHz to 0.5 THz.

### 3.1. E-Field and Power Density Radiation Patterns of Plane Waves

**Figure 3:**
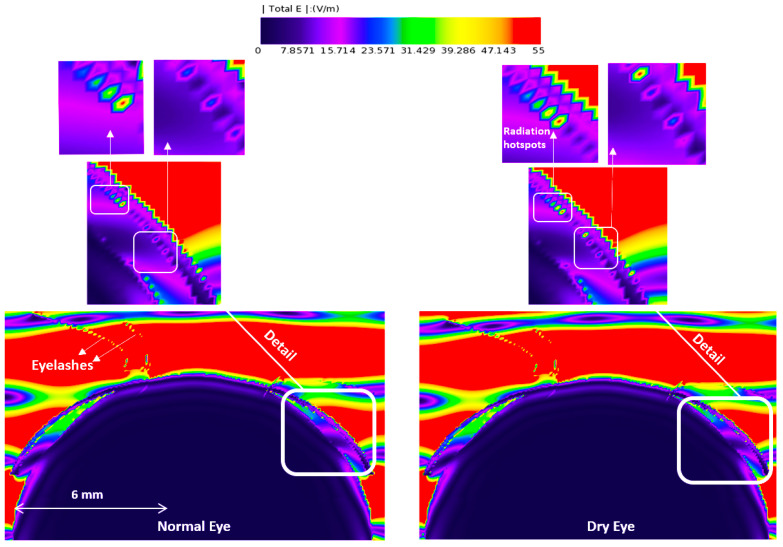
E-field distribution in the eyeball, a 30 GHz plane wave source was used with an incident power density of 20 W/m^2^. The E-field distribution was captured using a planar sensor bisecting the model in the sagittal plane.

**Figure 4:**
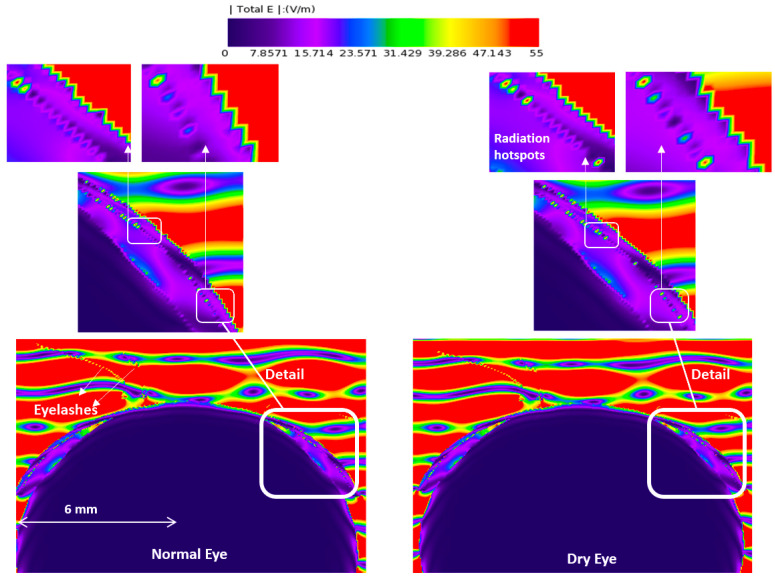
E-field distribution in the eyeball, a 60 GHz plane wave source was used with an incident power density of 20 W/m^2^. The E-field distribution was captured using a planar sensor bisecting the model in the sagittal plane.

**Figure 5:**
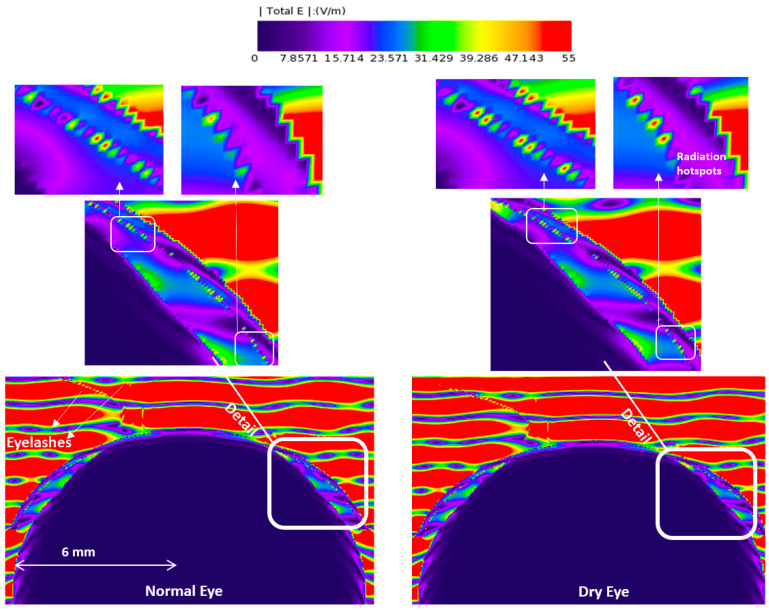
E-field distribution in the eyeball, a 90 GHz plane wave source was used with an incident power density of 20 W/m^2^. The E-field distribution was captured using a planar sensor bisecting the model in the sagittal plane.

**Figure 6:**
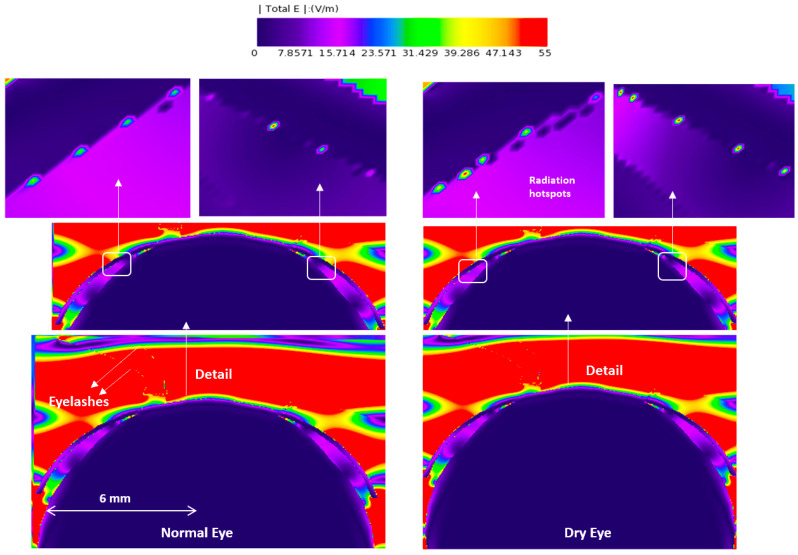
E-field distribution in the eyeball, a 500 GHz plane wave source was used with an incident power density of 20 W/m^2^. The E-field distribution was captured using a planar sensor bisecting the model in the sagittal plane.

**Figure 7:**
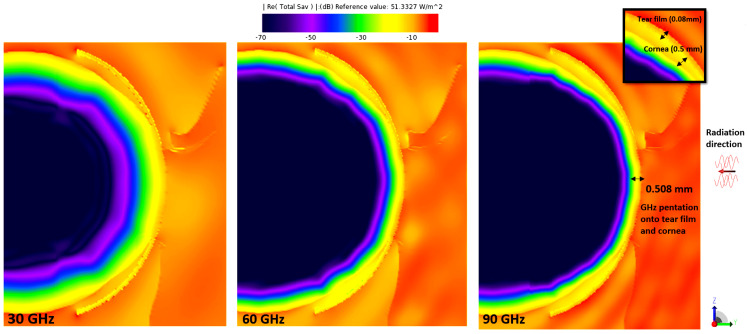
Power density distribution in the eyeball for radiation sources at 30, 60, and 90 GHz. Values are given in logarithmic scale (in dB).

**Figure 8:**
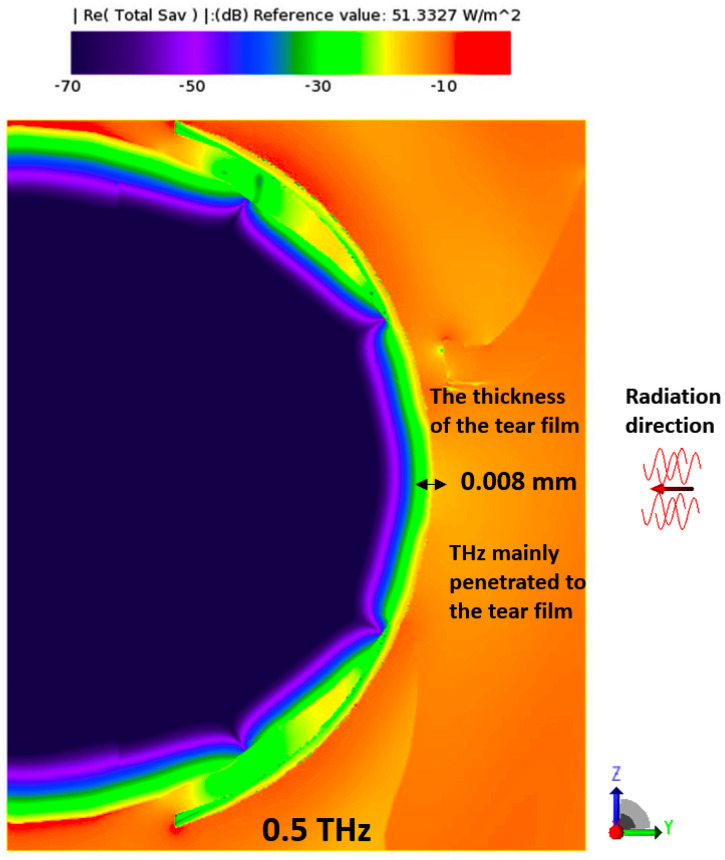
Power density distribution in the eyeball for radiation source at 0.5 THz. Values are given in logarithmic scale (in dB).

Figure 3, Figure 4, Figure 5 and Figure 6 depict the distribution of E-field strength for incident waves ranging from 30 GHz to 0.5 THz. Each figure includes two images, representing the interaction with normal and dry eyes, respectively. The power density distribution (in logarithmic scale) resulting from an incident wave from a plane wave radiation source at 30, 60, and 90 GHz is presented in Figure 7. Figure 8 represents the logarithmic scale of the power density distribution resulting from an incident wave from a plane wave radiation source at 0.5 THz. The colour levels in Figure 7 and Figure 8 correspond to the power density.

### 3.2. Temperature Rise

The comparison of temperature rises for three radiation sources at 30, 60, and 90 GHz is shown in Table 3. These were obtained by applying a thermal sensor in XFdtd. The thermal sensor captured the temperature rise in the cornea due to radiofrequency (RF) exposure (at 30, 60, and 90 GHz). This computation used a modified version of Pennes’ bio-heat transfer equation, accounting for conductive heat transfer between thermally connected tissues, perfusion impacts (the blood flow in the epidermis), metabolic processes, and general RF heating due to dissipated power.

## 4. Discussion

The results of the computer simulations make it possible to estimate the impact of tear film thickness and hydration on EM absorption by the cornea.

We conducted a comparison of temperature elevation in the cornea between dry and normal eyes when exposed to plane wave radiation sources with power densities of 20 Wm^−2^ and 100 Wm^−2^. Based on the findings presented in Table 3, it can be inferred that the tear film, in a normal eye configuration (e.g., with a thickness of 0.008 mm and water ratio of 80%), mitigates the heating of the cornea across all frequencies.

The increase in cornea temperature was 0.07216 °C in dry eye and 0.07078 °C in the normal eye from a 90 GHz plane wave radiation source in each case, with an incident power density of 100 W/m^2^. This represents a 1.95% increase in temperature elevation when the tear film is thinner. As shown in Table 3, the maximum temperature rises in the cornea always remained under 1 °C, even when the power density of the incident plane wave was set at 100 W/m^2^. Given that skin penetration depth is below 0.4 mm at frequencies above 100 GHz [18,40], the radiation is mainly absorbed by the eyelids (epidermis layer) at higher frequencies, resulting in less of a temperature rise in the cornea. Upon comparing the temperature rise in normal eyes to that in dry eyes, it was observed that a five-fold increase in incident power density led to a slightly higher temperature rise difference. This can be attributed to the variation in heat loss rates between high- and low-incident power sources. It should be noted that the two models used for normal eyes and dry eyes are not comprehensive enough to provide detailed insights into the severity of biological impacts caused by mmW and THz radiation. However, tear film thickness and hydration do influence the absorption of mmW and THz radiation by the cornea.

The E-field distribution of a plane wave source at 30 GHz to 0.5 THz are shown in Figure 3, Figure 4, Figure 5 and Figure 6. Some radiation hot spots are apparent in the case of dry eye at almost all frequencies, which illustrates the penetration depth is reached in the cornea and more radiation is absorbed by the cornea in the dry eye model (shown in Figure 3, Figure 4, Figure 5 and Figure 6 right panels). However, these hot spots were fewer for a normal eye. The main potential factor is variations in the thickness and hydration of the tear film. By demonstrating the presence of radiation hot spots and the differences between dry eye and normal eye models, this study contributes to the state-of-the-art knowledge regarding the localized distribution of EM fields in the eye at various frequencies.

In thick tear film, the E-field studies show that the thicker tear film with increased water content protects the underlying tissue (i.e., the cornea). These observations demonstrate that the tear film’s thickness and water content are the dominant factors determining radiation penetration into the corneal tissues.

As shown in Figure 7, with increasing plane wave frequency, the penetration depth becomes smaller than 600 µm, and most of the EM radiation is reduced to being deposited in the tear film and the cornea, in line with the previous statements on the penetration depth of the EM fields into biological tissues [19]. Figure 8 further supports these observations by showing that, at a frequency of 0.5 THz, most of the radiation is deposited in the tear film. This finding implies that reducing the thickness of the tear film could potentially increase the absorption of power in the cornea, particularly at higher frequencies. Compared to relevant literature, these findings corroborate previous studies that reported the limited penetration depth of mmW-THz fields into biological tissues. The specific depth of 600 µm mentioned in the present study aligns with the depth range observed in previous research. Moreover, identifying the tear film and cornea as significant sites of EM radiation absorption at higher frequencies is consistent with prior studies that highlighted the importance of these tissues in understanding the interaction between mmW-THz radiation and ocular structures.

As illustrated in Figure 7 and Figure 8, the power density in the structures posterior to the cornea (iris and lens) is much lower due to the attenuation in those tissues. The penetration depth due to EM exposure at 30 GHz to 0.50 THz ranges from a millimetre down to the low micrometer range.

Until now, only a few studies have provided comprehensive dielectric data for all ocular tissues, including the iris and lens, in the THz frequency range. The availability of accurate dielectric properties for tissue modeling is crucial, especially for supporting RF computational studies [12]. However, the limited availability of dielectric data at higher frequencies, such as the THz region, poses a significant challenge for RF computational simulation studies.

Additionally, when modeling higher frequencies, there is substantial demand for computational resources, and achieving accuracy at greater THz frequencies requires finer meshing. In our study, the computational time for a single simulation at 0.5 THz was approximately 24 h due to the complexity of the proposed multi-layer design. However, it is important to note that the anatomical details of the model may be too intricate for the available computational system. Therefore, a trade-off between anatomical accuracy and computational time must be carefully considered to ensure optimal computational studies of RF radiation.

In sum, the temperature of the cornea exhibited an increase of 0.07216 °C in the case of dry eye and 0.07078 °C in the normal eye when exposed to a 90 GHz plane wave radiation source, with an incident power density of 100 W/m^2^. This signifies a temperature elevation rise of 1.95% when the tear film is thinner. It is important to note the observed trend of increasing temperature elevation with higher frequencies, and it is crucial to emphasize the possibility of “slightly” more noticeable effects. However, to validate these phenomena and gain a better understanding of them, further in vivo investigation is required.

## 5. Conclusions

With the advances in 5G technology and the future development of potential 6G operating systems, there is growing anticipation for higher frequencies reaching up to 4 THz in the electromagnetic (EM) spectrum. With the increase in frequency, reaching significantly beyond 6 GHz, the penetration depth decreases, resulting in a more significant deposition of EM energy within superficial tissues with a high water content, such as the cornea. In this study, we analyzed the power density and electric field distributions and the resulting temperature rise in the cornea when exposed to plane waves ranging from 30 GHz to 0.5 THz. Our findings indicate that the impact of mmW and THz radiation on the cornea may be slightly more noticeable in the case of dry eyes. However, further investigation is needed through in vivo studies to gain deeper insights into these effects and their potential implications. 

## Figures and Tables

**Table 1:** Simulation parameters for thick and thin tear film models.

Model	Thick Tear Film			Thin Tear Film		
		Yee cell dimension			Yee cell dimension	
Frequency (GHz)	Time step (s)	Max cell size (mm)	Minimum cell size (mm)	Time step (s)	Maximum dimension	Minimum Dimension
30	3.92157 × 10^−14^	0.393 × 0.295 × 0.297	0.015 × 0.041 × 0.021	3.92157 × 10^−14^	0.393 × 0.295 × 0.297	0.015 × 0.041 × 0.021
60	3.40136 × 10^−14^	0.245 × 0.245 × 0.245	0.013 × 0.041 × 0.019	3.40136 × 10^−14^	0.245 × 0.245 × 0.245	0.013 × 0.041 × 0.019
90	3.38753 × 10^−14^	0.153 × 0.166 × 0.159	0.013 × 0.041 × 0.019	3.38753 × 10^−14^	0.153 × 0.166 × 0.159	0.013 × 0.041 × 0.019
500	2.55232 × 10^−14^	0.058 × 0.058 × 0.059	0.013 × 0.013 × 0.013	2.55232 × 10^−14^	0.058 × 0.058 × 0.059	0.013 × 0.013 × 0.013

**Table 2:** Thermal parameters and densities of the tissues incorporated in the model.

Tissue	Thermal Conductivity W/(m·K)	Specific Heat Capacity C J/(kg·K)	Density (kg/m^3^)
Cornea	0.58	4178	1050
Aqueous Humour	0.58	3997	996
Iris	0.52	3600	1050
Lens	0.4	3000	1000
Eyelash	0.37	1680	0.0013
Eyelids	0.36	3390	1109

**Table 3:** Comparison of temperature rises for normal and dry eyes with a plane wave source at densities of 20 Wm^−2^ and 100 Wm^−2^.

Normal Eye		Dry Eye		Temperature Rise Difference (%)
30 GHz		30 GHz		
Incident Power Density (Wm^−2^)	Maximum Temperature rise (°C)	Incident Power Density (Wm^−2^)	Maximum Temperature rise (°C)	
20	0.02557	20	0.02575	0.70
100	0.1278	100	0.1288	0.78
60 GHz		60 GHz		
Incident Power Density (Wm^−2^)	Maximum Temperature rise (°C)	Incident Power Density (Wm^−2^)	Maximum Temperature rise (°C)	
20	0.02475	20	0.0252	1.82
100	0.1238	100	0.1261	1.86
90 GHz		90 GHz		
Incident Power Density (Wm^−2^)	Maximum Temperature rise (°C)	Incident Power Density (Wm^−2^)	Maximum Temperature rise (°C)	
20	0.01412	20	0.01439	1.91
100	0.07078	100	0.07216	1.94
